# The progress of radiomics in thyroid nodules

**DOI:** 10.3389/fonc.2023.1109319

**Published:** 2023-03-07

**Authors:** XiaoFan Gao, Xuan Ran, Wei Ding

**Affiliations:** Department of Thyroid Surgery, The Second Hospital of Jilin University, Changchun, China

**Keywords:** radiomics, thyroid nodules, machine learning, artificial intelligence, PTC

## Abstract

Due to the development of Artificial Intelligence (AI), Machine Learning (ML), and the improvement of medical imaging equipment, radiomics has become a popular research in recent years. Radiomics can obtain various quantitative features from medical images, highlighting the invisible image traits and significantly enhancing the ability of medical imaging identification and prediction. The literature indicates that radiomics has a high potential in identifying and predicting thyroid nodules. So in this article, we explain the development, definition, and workflow of radiomics. And then, we summarize the applications of various imaging techniques in identifying benign and malignant thyroid nodules, predicting invasiveness and metastasis of thyroid lymph nodes, forecasting the prognosis of thyroid malignancies, and some new advances in molecular level and deep learning. The shortcomings of this technique are also summarized, and future development prospects are provided.

## 1 Introduction

The epidemiological characteristics and clinical examination methods of thyroid nodules: Thyroid nodules are widespread clinically, and the incidence continues to rise worldwide, with an autopsy study estimating that 50% to 60% of adults may have thyroid nodules (1, 2). High-resolution ultrasound (US) can detect thyroid nodules in 19%- 68% (3) of randomly selected individuals, of which thyroid cancer occurs in 7% to 15% (4). Thyroid cancer is the most common endocrine malignancy in the United States (5) and the fifth most common cancer among women (6). The benign thyroid nodules without surgical indications generally do not require special treatment. In contrast, malignant thyroid nodules should be elective surgical treatment once diagnosed, and neck dissection should be performed if lymph node metastases are present. Some patients need to be treated with Iodine-131 nuclide after the operation (7) and predict the prognosis. Papillary thyroid carcinoma (PTC) is the most common pathological type of thyroid cancer. It usually has a good prognosis, but relapse patients have a poor prognosis. About 10%-15% of PTC will relapse, and recurrent PTC has aggressive characteristics such as extra-thyroid extension (ETE), invasive cell subtypes, lateral neck lymphatic metastasis, resistance to therapy, and distant metastases (8). The challenge for clinicians is to balance treatment approaches so that patients with low-risk or benign thyroid nodules are not over-treated, while patients with high-risk or malignant thyroid nodules need more aggressive therapies. Therefore, the differential diagnosis of thyroid nodules and the risk stratification are essential and helpful for the subsequent individualized treatment.

Currently, ultrasound(US), computed tomography (CT), magnetic resonance imaging(MRI), and nuclear medicine imaging, such as positron emission tomography/computed tomography (PET/CT), are commonly used in the clinic to evaluate thyroid nodules (9). They are mainly used to assess the benignity and malignancy of nodules, the degree of invasion by adjacent tissues, and lymph node metastasis (10). Medical imaging has become routine clinical practice to provide information about the characteristics of human tissues in a non-invasive and repeatable manner (11). However, current risk stratification for diagnostic imaging of thyroid nodules is subjective. It relies heavily on the clinician’s empirical judgment, and there is a large amount of untapped digital information in various images. Many researchers have attempted to develop non-subjective methods, including artificial intelligence models, to mine previously unused data in pictures to help solve this problem.

## 2 Radiomics

### 2.1 The development of radiomics

The emergence of radiomics cannot be separated from the development of artificial intelligence and machine learning. Artificial intelligence has been developed rapidly with the rapid progress of computer hardware computing power and the continuous iteration of new algorithms. Machine learning, as an essential subset of artificial intelligence (12), generally uses computer language to deeply mine existing prior knowledge or data, learn the relationship between high-dimensional features and target variables based on training samples, continuously learn from the data, optimize the prediction process and improve performance through a series of statements, and build an accurate model to make accurate predictions. The technology of radiomics based on machine learning is getting more and more attention in clinics. Since the birth of artificial intelligence, humans have tried to apply it to medicine (13). Some early applications, such as Computer Aided Detection (CADe) and Computer Aided Diagnosis System (CADx), were developed to detect and diagnose abnormal areas in human tissues by analyzing image features (14). In recent years, computational analysis and artificial intelligence (AI) have played an increasingly important role in various acquisition and data processing aspects. For example, AI-based image reconstruction dramatically reduces the time required for image reconstruction, producing images of comparable quality while providing the ability to reconstruct large datasets in real time. In addition, the availability of artificial intelligence lays the foundation for automated image post-processing, including segmentation and volumetric analysis.

### 2.2 The definition of radiomics

The term “radiomics” was first proposed by Dutch scholar Philippe Lambin et al. in 2012 (15). It is defined as extracting high-throughput features from medical images, using automatic or semi-automatic analysis methods to conduct deeper data mining of image information, and associating it with other clinical data for evidence-based clinical diagnosis and treatment decision support. Compared with traditional manual interpretation, radiomics is more objective, has higher information utilization, better interpretation repeatability, and more accessible quantitative analysis and knowledge and experience inheritance. With the rise of precision medicine worldwide, radiomics provide clinicians with new tools and means to achieve more accurate and personalized diagnosis and treatment of patients.

### 2.3 Radiomics workflow

**Figure 1:**
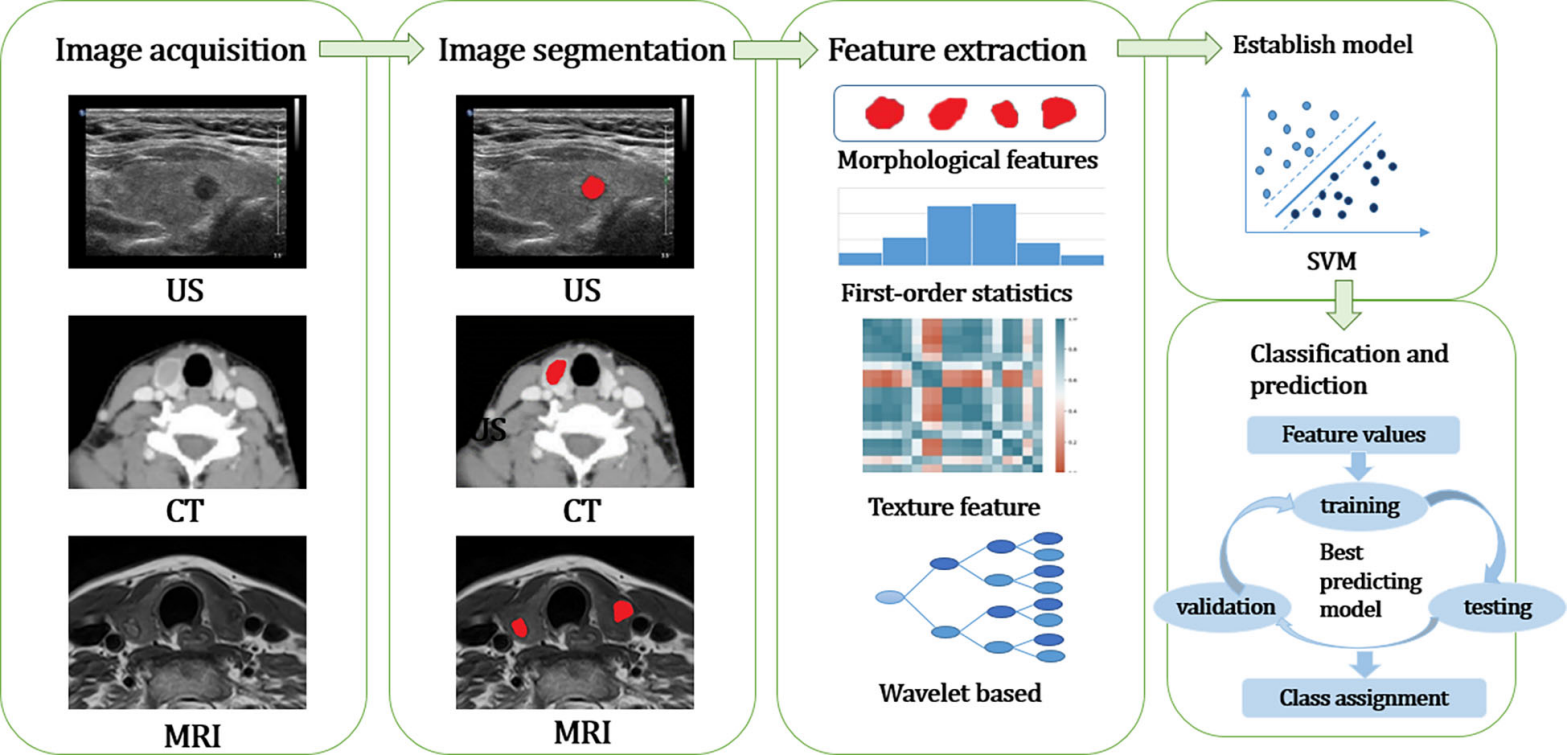
The workflow of radiomics can be summarized as follows: ① Image acquisition; ② Image segmentation; ③ Feature extraction and Feature selection; ④ Establish models and databases to classify the prediction results. US, ultrasound; CT, computed tomography; MRI, magnetic resonance imaging; SVM, support vector machine.

The workflow of radiomics can be summarized as follows: ① Image acquisition; ② Image segmentation; ③ Feature extraction; ④ Feature selection; ⑤ Establish models and databases to classify the prediction results (**Figure 1**).

#### 2.3.1 Image acquisition

Image acquisition is mainly carried out by CT, MRI, PET-CT, and other image scanning methods. When collecting images, the same or similar scanning machine should be selected as far as possible and appropriate parameters should be set, such as proper layer thickness and pixel size.

#### 2.3.2 Image segmentation

Image segmentation is crucial in radiomics because many extracted features may depend on the segmented region. Before segmentation, the images were reviewed carefully, high-quality images were selected, and artifacts were eliminated. Two experienced physicians work together to verify the location and area of the nodules on the chosen high-quality images and to outline the region of interest (e.g., tumor) as the region of interest (ROI). When delineating, try to cover all tumors and avoid normal thyroid tissue. There are three methods of image segmentation: manual segmentation, semi-automatic segmentation, and automatic segmentation.

Manual segmentation is susceptible to subjective influence related to operator experience. It requires a large amount of source data to establish a radiomics database, so it is time-consuming and labor-intensive to use manual segmentation to complete this work. Fully automatic segmentation refers to using computer algorithms to outline the nodes without human involvement, with less workload, which can, to some extent, compensate for the poor repeatability of manual segmentation but is prone to misjudge the boundaries of specific nodes. Semi-automatic segmentation means that the computer automatically draws the outline of the nodes and then uses manual operation to make corrections based on experience. This method reduces the workload and can also compensate for the defects of fully automatic segmentation that may misidentify the node boundaries. Evaluating by multiple clinicians or a combination of algorithms can be considered to avoid possible prejudice.

#### 2.3.3 Feature extraction and selection

According to the number of voxels involved in the calculation, the extracted features can be divided into shape features, first-order features, second-order features, and higher-order features (16).

Morphological features, i.e., based on the geometric characteristics of the ROI, such as volume, maximum surface area, and maximum diameter. First-order features: depend on the distribution of gray-scale intensities within the ROI, without considering the spatial relations within the ROI, for example, gray-scale mean, maximum value, minimum value, standard deviation, variance, mean absolute deviation, variance, skewness, sharpness, energy, entropy, etc. Second-order features, also known as texture features (17), quantify the heterogeneity within a tumor and explain the spatial dependence or co-occurrence of information between neighboring voxels. Texture features are not computed directly from the original image but from different description matrices encoded by the specific spatial relationships between pixels or voxels in the original image. In the original image, there are some spatial relationship matrices among the intensities of the encoded images from which a large number of texture features can be computed. For example, the absolute gradient, the gray-scale co-generation matrix (GLCM), the gray-scale run length matrix (GLRLM), the gray-scale size region matrix (GLSZM), and the gray-scale distance region matrix (GLDZM). Higher-order features are computed after applying mathematical transforms and filters highlighting repeating patterns, histogram-oriented patterns, or local binary patterns, such as wavelet or Fourier transforms. With the development of radiomics and to standardize terminology, the Image Biomarker Standardization Initiative 2020 (IBSI) provides guidelines for the accurate naming, definition, and reporting of imaging histology features (18).

#### 2.3.4 Feature selection

After extracting the high-latitude features, it is necessary to eliminate unreliable, under-informed, or redundant features to avoid dimensionality catastrophe and over-fitting of the model, improve learning accuracy, and reduce computation time. This step is called data dimensionality reduction, also called data cleaning, data selection. The last absolute shrinkage and selection operator (LASSO) Cox regression model, maximum relevance and minimum redundancy (mRMR), and principal component analysis (PCA) are the commonly used feature reduction methods.

#### 2.3.5 The model algorithm was established for classification and prediction

Other information can combine the above features with building models and databases to develop classifiers to predict results, which is the ultimate goal of radiomics (19). At present, the commonly used modeling machine learning algorithms in radiomics are as follows (20): Logistic regression, random forest (RM), support vector machine (SVM), Decision Tree, k-Nearest Neighbor(KNN), artificial neural networks (ANNs), Bayesian algorithm (Bayes), clustering algorithm (such as K-means/DBSCN). All of them are supervised learning algorithms except the clustering algorithm, which is unsupervised learning (21). The implementation of the classification model involves at least two stages: training and testing. The training phase is learning the classification model itself, and the training data must be large enough and representative of the general population. The test phase is to use or test the model learned in the training phase on new samples, and the data used in this phase is called test data. A third phase, the validation phase, can be introduced to improve learning performance when sufficient samples are available. In this phase, the model parameters learned in the training phase are adjusted and optimized, which may include the number of variables used or their relative weights. The data used in this phase is called validation data (22).

## 3 Application of radiomics in the diagnosis and treatment of thyroid nodules

### 3.1 Differentiate benign and malignant thyroid nodules

#### 3.1.1 Ultrasound for differentiating benign and malignant thyroid nodules

With the continuous improvement of ultrasound instruments, the application of high-frequency ultrasound to small organs has become an essential part of non-invasive ultrasound diagnosis (23). Because of its high sensitivity, non-radioactivity, simple operation, and rapid diagnosis, it is the first choice for the clinical screening of thyroid nodules. The 2015 guidelines of the American Thyroid Association (ATA) emphasize the significance of ultrasound in detecting thyroid nodules (24). In recent years, some studies have proposed new ultrasound techniques such as contrast-enhanced ultrasound (CEUS) and especially US-elastography (USE) to improve thyroid nodule diagnosis accuracy greatly (25). Most studies have focused on developing a radiomics score for predicting thyroid malignancy using ultrasound images and investigating it as a complementary tool to improve the performance of risk stratification systems. Jinyu Liang (26) used ultrasound images to perform radiomics scores and compared them with ACR TI-RADS scoring standards. The ultrasound radiomics score showed good discriminative and predictive value, and decision curve analysis showed that the model using the radiomics score added more benefits than the ACR score model for junior radiologists. Jiyoung Yoon (27) used multivariate logistic regression analysis to establish two prediction models: one based on clinical variables and the other using clinical variables combined with a radiomics score. The results showed that: The AUC of the prediction model composed of clinical variables and radiomics score was significantly higher than that of the model consisting of clinical variables alone (0.839 vs. 0.583). ShiYanping (28) has also conducted a similar experiment, and Shi established three models: the model of clinical imaging group learning model, the clinical imaging group joint model, and combined images with nomogram omics. Results show that the clinical imaging omics collaborative model is higher than the above two models, has a predictive value, and has a higher net efficiency.

#### 3.1.2 MRI for differentiating benign and malignant thyroid nodules

MRI has a high contrast resolution, can provide excellent soft tissue contrast, and can be all-round, multi-angle, and multi-plane to discriminate evaluation of nodules for benign and malignant differentiation (29), while no radiation damage compared with CT. In recent years, MRI has been shown to accurately stage cancer and support patient follow-up in some areas, especially colorectal cancer, prostate cancer, and gynecological tumors (30). MRI is the most sensitive imaging method to diagnose early liver and metastatic brain diseases. It is also routinely used to determine the extent of bone marrow involvement and determine the lesions in bone malignancies. However, due to its long operation time, motion artifacts and other problems are rarely used to diagnose thyroid nodules. Wang (31) used high b-value diffusion-weighted imaging (DWI), PCA, and PCC for dimensionality reduction and constructed ten models: support vector machines (SVM), Latent Dirichlet Allocation (LDA), Auto-Encoder (AE), Random Forests (RF), Logistic Regression (LR), Least Absolute Shrinkage and Selection Operator (LASSO), decision tree, gene programming, Naive Bayes. They used sensitivity, specificity, accuracy, and AUC four indexes for verification. When “NormUnit” was used for standardization, principal component analysis was used for dimensionality reduction, ANOVA was used for eigenvalue screening, and 15 eigenvalues were selected; the differential diagnosis effect of the model was the best, the accuracy was 85.71%, and the sensitivity was 80.00%. The specificity was 100.00%, and AUC was 0.925. Xia Liang (32) selected T2 weighted images and apparent diffusion coefficient images. They used factor analysis to screen features and then further screened from the above features to build an SVM model. The model’s accuracy was 88%, the sensitivity was 98%, the specificity was 80%, and the area under the curve was 0.92. A total of 15 features were screened, divided into general, morphological, and grey spatial distribution-related features (including GLZML, GLRLM, etc.). From the feature importance of the support vector machine model with polynomial kernel function, CONVENTIONAL_Q2 characteristics in ADC images, i.e., the second quarter number (median), and CONVENTION‐ AL_Q3, i.e., the third percentile, are of great significance for the recognition of thyroid papillary carcinoma. At the same time, CONVENTIONAL_std-t is the most important newly discovered characteristic of T2WI to differentiate nodular goiter from thyroid papillary carcinoma.

#### 3.1.3 CT for identification of benign and malignant thyroid nodules

Thyroid imaging studies are mostly based on ultrasound, with fewer studies on CT and MRI. Still, thyroid nodules are usually first found on other cross-sectional patterns of computed tomography (CT) and magnetic resonance imaging (MRI) (33). Thyroid lesions can be considered incidental findings, with thyroid nodules found in about 16% of chest CT scans. Enhanced CT scans can show the characteristic enhancement of the thyroid, nodules, and surrounding tissues, which is of great significance for the qualitative diagnosis of thyroid nodules. The use of CT images can avoid the bias of ultrasonic images due to subjective operation, the image standardization is higher, and the trained model has a stronger generalization ability. Wu Yuqiang (34) identified benign and malignant thyroid nodules based on enhanced arterial phase single-layer CT images and excluded calcification and cystic necrosis lesions. It is found that GlCM-based texture feature values (entropy and FD) differ significantly between benign and malignant nodules, and the sensitivity and specificity are higher when the entropy boundary value is 5.00. However, Guo Wei (35) did not avoid cystic necrosis and calcification in nodules when sketching areas of interest with single-layer CT-enhanced images. The results showed that the differential diagnosis efficiency was better when entropy 6.09 was used as the boundary value. Entropy is the most objective texture parameter feature, reflecting the complexity and heterogeneity of the internal structure of the tumor. The pixel value represents the number of pixels contained in the image. The higher the value, the clearer the image will be. Skewness, peak state, and standard deviation reflect the distribution of pixel values. Using the volume measurement method, Hu Yunting (36) extracted the 3D texture feature values of enhanced CT images of 41 thyroid nodules, including skewness, kurtosis, entropy, inhomogeneity, standard deviation, and average intensity. Among them, the difference in entropy between benign and malignant thyroid nodules was obvious; the entropy value > 3.79 indicated that thyroid nodules were more likely to be malignant. Zhang Dawei (37) collected and analyzed 203 patients with thyroid micronodules and used LASSO Logistic dimension reduction to analyze and compare six models: Forest, SVM, KNN, Tree, Bayes, and Logistic. Accuracy, specificity, sensitivity, and AUC of differential diagnosis of benign and malignant thyroid micronodules by different models. It is concluded that the enhanced CT image based on the Forest model has the best diagnostic efficacy for benign and malignant thyroid nodules. Du Dandan (38) used the same method to identify thyroid adenoma and papillary thyroid carcinoma more prominent than 1cm. Six radiomics models were constructed, including Forest, SVM, KNN, Tree, Bayes, and Logistic. The accuracy, specificity, and sensitivity of each model in the differential diagnosis of benign and malignant thyroid micronodules were higher than the results of conventional enhanced CT studies. The plain CT and improved Forest imaging model had a higher value in the differential diagnosis of PTMC and MNG. The above studies discussed the feasibility of establishing an imaging omics model based on existing CT images of patients according to the model’s efficacy to increase better clinicians’ diagnostic accuracy in differentiating benign and malignant thyroid nodules and maximize the value of thyroid CT examination.

### 3.2 Prediction of invasion and thyroid lymph node metastasis

Although PTC is considered an indolent tumor, some cancer cells will metastasize to lymph nodes around the thyroid gland (39, 40), mainly including central lymph node metastasis and lateral neck lymph node metastasis. However, excessive lymph node dissection will lead to many complications. The greater the scope of surgery, the greater the likelihood of complications such as damage to the supraglottic nerve and recurrent laryngeal nerves and the paramedian nerves, permanent hypoparathyroidism, celiac fistula, and so on (41). Therefore, the preoperative judgment of LNM metastasis is an important indicator for the prognosis, surgical scope, and surgical method of thyroid cancer. Accurate preoperative diagnosis of thyroid lymph node metastases is crucial to determining staging and individualized treatment plans (42, 43).

#### 3.2.1 Ultrasound for predicting invasivity and thyroid lymph node metastasis

There are two ways to predict lymph node metastasis in the neck of the thyroid gland. One is based on the image of the primary lesion, and the other is based on the image of the metastatic lymph node. Although it is more direct to predict the invasiveness of papillary thyroid carcinoma by extracting radiomics characteristics of metastatic lymph nodes, the detection rate of metastatic lymph nodes is low, and there are limitations. Therefore, most prediction models predict the invasiveness based on the ultrasound radiomics characteristics of the primary PTC lesion. The accuracy, sensitivity, and specificity of Zhou Shichong et al. in determining metastatic lymph nodes based on the characteristics of PTC primary lesions were 0.731, 0.714, and 0.74, which were much higher than the diagnostic rate of two-dimensional ultrasound in conventional studies (44).Some researchers have further analyzed ultrasound radiomics combined with nomograms. Xian Wang (45) first divided patients into the ETE (extent-extra-thyroid extension (ETE) group and non-ETE group according to pathological results, established a radiomics nomogram and evaluated its accuracy and clinical practicability. Decision curve analysis showed that the nomogram of ultrasound radiomics has good clinical application value. Yuyang Tong (46) used the same method to construct a nomogram based on metastatic lymph node images. It concluded that radiomics features were significantly correlated with lateral neck lymph node metastasis in the two groups (p<0.001). The training and validation sets show good recognition and calibration ability, and the AUC is 0.946 and 0.914, respectively.

#### 3.2.2 MRI for the prediction of invasiveness and thyroid lymph node metastasis

MRI is also advocated for cervical and mediastinal lymph node imaging. It can be performed with or without the injection of gadolinium chelate as a contrast agent and without injecting any iodine contrast agent. In many thyroid cancer patients, the performance of MRI on neck and mediastinum imaging has not been directly compared with CT. It can paint a better picture of anything involving the airway than a CT scan. It is often used as a second-line imaging technique in patients with CT scans showing or suspected lesions to characterize these lesions better (3, 47). There are many studies on the prediction of cervical lymph node metastasis at the MRI level, most of which are for the prediction of central lymph node metastasis. However, some early studies only used texture for statistical analysis, such as Zhang Heng (48) used the texture analysis method of the first-order histogram and second-order GLCM to analyze the texture of images at the T2WI stage and extract 9 texture parameters. He found that entropy, standard deviation, correlation, and the angular second-order moment significantly differed among PTC patients with or without cervical lymph node metastasis. Entropy reflects the non-uniformity of image texture, and the more complex the texture is in an image, the larger the entropy is. The angular second-order moment, also known as energy, is the sum of squares of GLCM element values, reflecting the uniformity of image grey distribution and texture thickness. The larger the value, the more uniform and less heterogeneous the image. Yao Xihu (49) used a similar method to analyze with a more significant sample number, delineated at the T2 stage, and selected eight texture parameters, among which entropy, angular second moment, and correlation were statistically significant except for standard deviation. The conclusion was consistent with ZhangHeng’. Hui Qin (50) selected image omics features from 109 adipose inhibition T2-weighted MRI images, determined the optimal features by the spearman correlation test, hypothesis test, and random forest method, and constructed eight prediction models. The model’s validity was verified by receiver operating characteristic (ROC) curve analysis. Finally, it is concluded that the combined model has better diagnostic efficacy in evaluating PTC lymph node metastasis.

#### 3.2.3 CT for predicting invasive and thyroid lymph node metastasis

Cross-sectional imaging studies such as CT and MRI also help physicians to evaluate lymph node metastases before surgery, especially in areas that are difficult to evaluate with ultrasound (e.g., posterior pharynx, mediastinum, and low-level IV lymph node sites). The surgeon must know the lesion site, morphological density, capsule invasion, and lymph node metastasis of the thyroid nodule (10). Shen Shasha (51) used wavelet transform technology to analyze CT venous phase images of thyroid cancer. The results showed that the sensitivity of the training group and the verification group to predict central lymph node metastasis was 62.84% and 64.95%, respectively. And SMALL AREA LOW GREY LEVEL EMPHASIS (SALGLE) can be used as an independent predictor of risk factors. Su (52) retrospectively analyzed arterial and venous CT-enhanced images of 27 patients with lateral neck lymph node metastasis and 32 patients with non-lateral neck lymph node metastasis of thyroid cancer and adopted ROC curve analysis and multiple Logistic regression analysis. Based on histogram analysis and grey co-occurrence matrix (GLCM), the research results showed that Kurtosis had the best diagnostic area under the curve (0.884) and specificity (92.59%). Conversely, the average grey intensity had the best diagnostic sensitivity (90.62%). Arterial stage mean grey intensity (P=0.006, OR=24.297) and venous stage kurtosis (P=0.014, OR=19.651) were independent predictors of cervical lymph node metastasis. Hejunlin (53) retrospectively studied the plain and enhanced CT images of 197 patients with PTC and selected 107 features of plain, arterial and venous phases. SelectKBest in Python was used to construct the random forest algorithm. It is concluded that the imaging features of the above three stages can predict CLNM, and the prediction performance of the plain scan stage is better than that of the arterial and venous phases. ZhaoHongbo (54) retrospectively analyzed 173 lymph nodes by plain CT and double-phase enhanced CT. Of these, 89 were transferred, and 84 were not. He adopted the R language algorithm built into Darwin’s scientific research platform. He selected six features of the arterial phase and five features of the venous phase for algorithm analysis, which showed the best efficiency. Y.Zhou (55) retrospectively analyzed the dual-energy CT iodinogram (DECT) of 255 lymph nodes, of which 143 were non-metastatic and 112 were metastatic. By using LASSO dimension reduction, Logistic modeling, and nomogram decision analysis, he concluded that DECT imaging analysis was superior to CT imaging features in preoperative diagnosis of cervical lymph node metastasis in PTC patients.

### 3.3 Molecular level

There are few studies on ultrasonic radiomics at the molecular level. Zhou Shichong’s research (56) shows that the changes in protein molecules likely constitute the molecular basis of the differences in radiomics characteristics. Luo Peng’s study further proved that immunohistochemical markers cytokeratin 19 (CK-19), galectin-3 (Gal-3), thyroid peroxidase (TPO), and high molecular weight cytokeratin (HMWCK) play an essential role in the molecular diagnosis of thyroid nodules. Gu’s study (57) showed that the accuracy of thyroid peroxidase and galectin-3 prediction models were 81.4% and 82.5% in the training cohort and 84.2% and 85.0% in the validation cohort, respectively. As a diagnostic marker, the Gal-3 protein has been shown to play a fundamental role in thyroid carcinoma (58). In papillary cancer cells, the expression of antisense oligonucleotides was inhibited, and the malignancy was significantly reduced, while normal thyroid cells transfected with Gal-3 cDNA acquired a malignant phenotype (59, 60). The depth of this research provides a promising approach for future research.

### 3.4 Deep learning

In terms of deep learning, Hui Zhou (61) designed the basic convolutional neural network (CNN) model, transfer learning (TL) model, and a newly designed model named thyroid deep learning Radiomics (DLRT), and compared with radiologists; the results showed that the AUC of the above three radiomics models was greater than that of radiologists. The overall performance of DLRT is the best. Ilah Shin (62) developed two models: artificial neural network (ANN) and support vector machine (SVM) based classifier models. Compared with radiologists, the three models’ sensitivity, specificity, and accuracy were 32.3%, 90.1%, and 74.1%, respectively. 41.7%, 79.4%, and 69.0%. And 24.0%, 84.0%, and 64.8%.

### 3.5 The prognosis

Because PTC is often associated with good long-term mortality, making disease-free survival for recurrent or persistent disease the focus and endpoint of risk stratification, rather than mortality, would help personalize treatment and management to benefit more patients. Although promising, these results are preliminary and require further validation on larger, independent data sets before clinical use.

## 4 Common problems of radiomics and improvement methods

Most radiomics studies are retrospective, single-center, and small-sample studies In the future, prospective, multicenter, and large-sample studies are needed to verify its feasibility and serve as a clinical diagnostic tool to help diagnose thyroid nodules (63).

Most studies only divide ROI on the maximum cross-section of nodules, and the heterogeneity analysis of the whole tumor needs to be strengthened. Therefore, in further research, we should adopt the segmentation method with good repeatability and high reliability. Recently, researchers have used three-dimensional texture analysis, which maps the lesions in each layer of the tumor to make up the VOI of the tumor. This method avoids the subjectivity of physicians to a large extent, and the measured values are more stable, fully reflecting the heterogeneity of tumors. Studies have shown that ADC values calculated by full-volume ROI have higher diagnostic efficiency than those calculated by single-layer solid component ROI.

### 4.1 Lack of unified standards for radiomics steps

Image acquisition, image due to different hospital equipment including ultrasound, CT, MRI, and PET - CT model, parameters, and thus will be affected by the corresponding input data. Image group research needs in numerous hospitals data find strictly conform to the data into a set of conditions to ensure consistency, and doing so will lead to reducing the amount of data. Therefore, radiomics research needs to find a compromise point between the data volume and the inclusion specification to guarantee the essential data volume and support the study of large samples, multi-features, multi-sequences, and multi-methods. Therefore, all clinical and scientific researchers must collaborate to establish an open scientific research database. The Databases can link massive amounts of radiomics data from millions of patients (hopefully with all other relevant data) to form vast networks of fast learning. Still, there are also considerable data management hurdles (64).

### 4.2 Black box problem

Trained algorithm models usually have high accuracy, but due to the complexity of algorithm data, we cannot have an in-depth understanding of the inner work of these models, and there are unexplained black box problems (65). Traditional medical decisions are based on the knowledge of pathophysiology, but without understanding the underlying principles, clinicians and patients find it difficult to accept them. In addition, without biological experiments and clinical studies, it is difficult for regulators to approve the application of AI-aided diagnostic tools. The model lacks interpretability and cannot estimate the importance of each feature to its prediction result, let alone explain whether there is an interactive relationship between individual elements. Therefore, there is still room for improvement in applying artificial intelligence. It cannot wholly replace physicians in the process of diagnosing and treating patients. The black box algorithm’s opaqueness is combined with many ethical issues, which has become a hot research issue (66).

## 5 Summary and prospect

Although radiomics still faces many problems before it is widely used in clinical practice, the advantages of high reproducibility and easy implementation of radiomics indicate that it has great potential in clinical application. Radiomics can provide more guidance for the diagnosis, treatment, and prognosis of thyroid diseases by in-depth mining image information. As an emerging research method, it is an essential direction for the future development of radiomics to find a validated and reliable algorithm after repeated testing and refining with prospective, multi-centre, and large samples, which lays a solid foundation for precision medicine.

## Author contributions

WD proposed the research topic and guided support. XG designed the paper framework, drafted the paper, and revised the paper. XR collated relevant literature. All authors contributed to the article and approved the submitted version.
